# A deep‐learning model for one‐shot transcranial ultrasound simulation and phase aberration correction

**DOI:** 10.1002/mp.70259

**Published:** 2025-12-31

**Authors:** Kasra Naftchi‐Ardebili, Karanpartap Singh, Gerald R. Popelka, Kim Butts Pauly

**Affiliations:** ^1^ Department of Bioengineering Stanford University Stanford California USA; ^2^ Department of Electrical Engineering Stanford University Stanford California USA; ^3^ Departments of Otolaryngology and Radiology Stanford University Stanford California USA; ^4^ Departments of Radiology, Bioengineering, and Electrical Engineering Stanford University Stanford California USA

**Keywords:** acoustic simulation, Deep learning, transcranial ultrasound

## Abstract

**Background:**

Transcranial ultrasound is a promising non‐invasive neuromodulation technique with applications, including neuronal activity modulation, blood–brain barrier opening, targeted drug delivery, and thermal ablation. Its ability to deliver focused ultrasound waves to precise brain regions has led to over 50 clinical trials targeting conditions such as opioid addiction, Alzheimer's disease, dementia, epilepsy, and glioblastoma. However, skull heterogeneity complicates accurate focal spot prediction and energy delivery, requiring rapid yet precise phase aberration correction in clinical workflows.

**Purpose:**

To address the trade‐off between computational efficiency and accuracy in current focus prediction methods, we introduce TUSNet, a deep learning framework for rapid and accurate transcranial ultrasound pressure field and phase aberration correction computation.

**Methods:**

TUSNet, an end‐to‐end neural network, was trained to predict both 2D transcranial ultrasound pressure fields and phase corrections. TUSNet was trained on 180432 synthetic skull Computed Tomography (CT) segments, and tested on 1232 real skull CT segments. Its performance was benchmarked against k‐Wave, a MATLAB‐based acoustic simulation package, evaluating computation speed, focal spot accuracy, phase correction accuracy, and pressure magnitude estimation.

**Results:**

TUSNet computed pressure fields and phase corrections in 21 ms, which is over 1200× faster than k‐Wave, while achieving 98.3% accuracy in peak pressure magnitude estimation and a mean focal positioning error of only 0.18 mm relative to k‐Wave ground truth. End‐to‐end training took approximately 8 h on 4x NVIDIA A100 80 GB GPUs.

**Conclusions:**

TUSNet demonstrates that deep learning can provide accurate and rapid estimates of phase aberrations and transcranial pressure fields, offering a promising direction for accelerating ultrasound treatment planning. While the present validation is based on simulated, noise‐free ultrasound fields, the results establish a foundation that future experimental studies can build on to assess performance under real‐world clinical conditions.

## 1 INTRODUCTION

In recent years, transcranial ultrasound has been utilized for research and clinical applications, owing to its non‐invasive approach to neural tissue modulation, blood–brain barrier opening, drug delivery via nanoparticles, and thermal ablation. With its roots dating back to the pioneering work of Fry et al.,^1^ transcranial ultrasound has since been a focal point of interest within the research community, leading to many key advancements in the field and allowing safe and effective treatment in humans.^2^, ^3^, ^4^, ^5^, ^6^, ^7^, ^8^, ^9^, ^10^, ^11^, ^12^, ^13^, ^14^, ^15^, ^16^, ^17^, ^18^, ^19^ Moreover, at the moment, there are upwards of 50 ongoing clinical trials to assess the effectiveness of transcranial ultrasound in the noninvasive treatment of a range of disorders, including epilepsy, opioid addiction, Alzheimer's disease, glioblastoma, attention deficit hyperactivity disorder, and obsessive compulsive disorder.^20^ Despite these remarkable strides, the optimization of transcranial ultrasound parameters for individualized treatments remains a formidable challenge due to the complexity of ultrasound propagation in the human skull.^21^, ^22^, ^23^

The successful application of transcranial ultrasound relies heavily on the precise computation of the location and pressure at the focal spot. Ideal precision in transcranial ultrasound is on the sub‐millimeter order, and depends on the target size, intended outcome, and transducer frequency, but the obtained precision in focal positioning and energy deposition is greatly influenced by the skull's heterogeneity.^24^, ^25^, ^26^ The skull introduces phase aberrations to the ultrasound wavefront, which affect the location and intensity of the ultrasound focus within the brain, potentially leading to off‐target effects or reduced efficacy. Physics‐based simulation methods, such as the k‐space pseudospectral method^27^ and hybrid angular spectrum (HAS) method,^28^, ^29^ provide powerful tools to address this challenge by modeling the acoustic propagation of the ultrasound waves through the skull. Notably, for clinical relevance, accurate computation of the focal spot must be available within seconds, as clinicians rely on rapid feedback to iteratively adjust transducer position, steering, and acoustic power during targeting. Any delay beyond this short window disrupts this intraoperative workflow and makes real‐time correction impractical. However, these physics‐informed simulations are computationally intensive: resolving MHz‐range ultrasound propagation through a heterogeneous, high‐contrast medium requires fine spatial grids, Courant–Friedrichs–Lewy‐limited temporal steps, and often multiple forward–backward solves, causing runtimes to range from minutes to hours even on modern GPUs.^29^ This computational burden hinders their use in real‐time clinical settings.

Currently, commercial devices often rely on faster methods like ray tracing to predict the focus location and pressure. While these methods are computationally efficient, they may not always achieve the high level of accuracy needed for optimal transcranial ultrasound delivery.^29^ Leung et al. showed that while the InSightec Exablate 4000 was capable of computing the phase corrections for all of its 1024 elements in approximately 2 s, it only recovered 71±15% of the pressure at the target (compared against hydrophone ground truth measurements), while incurring a positioning error of 0.72±0.47mm. On the other hand, HAS recovered 86±5% of the pressure at the target, and at the same time reduced the positioning error down to 0.35±0.09mm. However, this improvement in targeting efficacy and accuracy came at a cost: what took InSightec's proprietary ray tracing 2 s to compute, took HAS 30 min.^29^ With the suboptimal positioning accuracies achievable with fast but less accurate methods such as ray tracing, clinicians are forced to iteratively adjust the position of the transducer on the patient until they see the intended focal spot using imaging methods such as magnetic resonance thermometry.^30^, ^31^ The ideal, which remains unresolved to this date, is to reach the accuracies achieved by HAS,^29^ but with run times similar to that of ray tracing.

Therefore, a major gap remains between the need for rapid and accurate predictions of the transcranial ultrasound pressure field and the currently available computational methods. While machine learning is poised to address this inherent trade‐off between accuracy and efficiency, few such attempts have been made to date. Shin et al.^32^ developed a super‐resolution neural network that transformed 1‐mm low‐resolution voxels into 0.5‐mm higher‐resolution voxels, improving the simulation run time by a remarkable 86.91%, but it did not correct for phase aberrations and utilized a single‐element transducer. Choi et al.^33^ proposed an interesting model that identified the ideal x,y,z location for a single‐element transducer placement, given a desired pressure field. However, this approach relied on prior knowledge of the desired transcranial pressure field, and the model was not tasked with computing and correcting for the phase aberrations.

**FIGURE 1:**
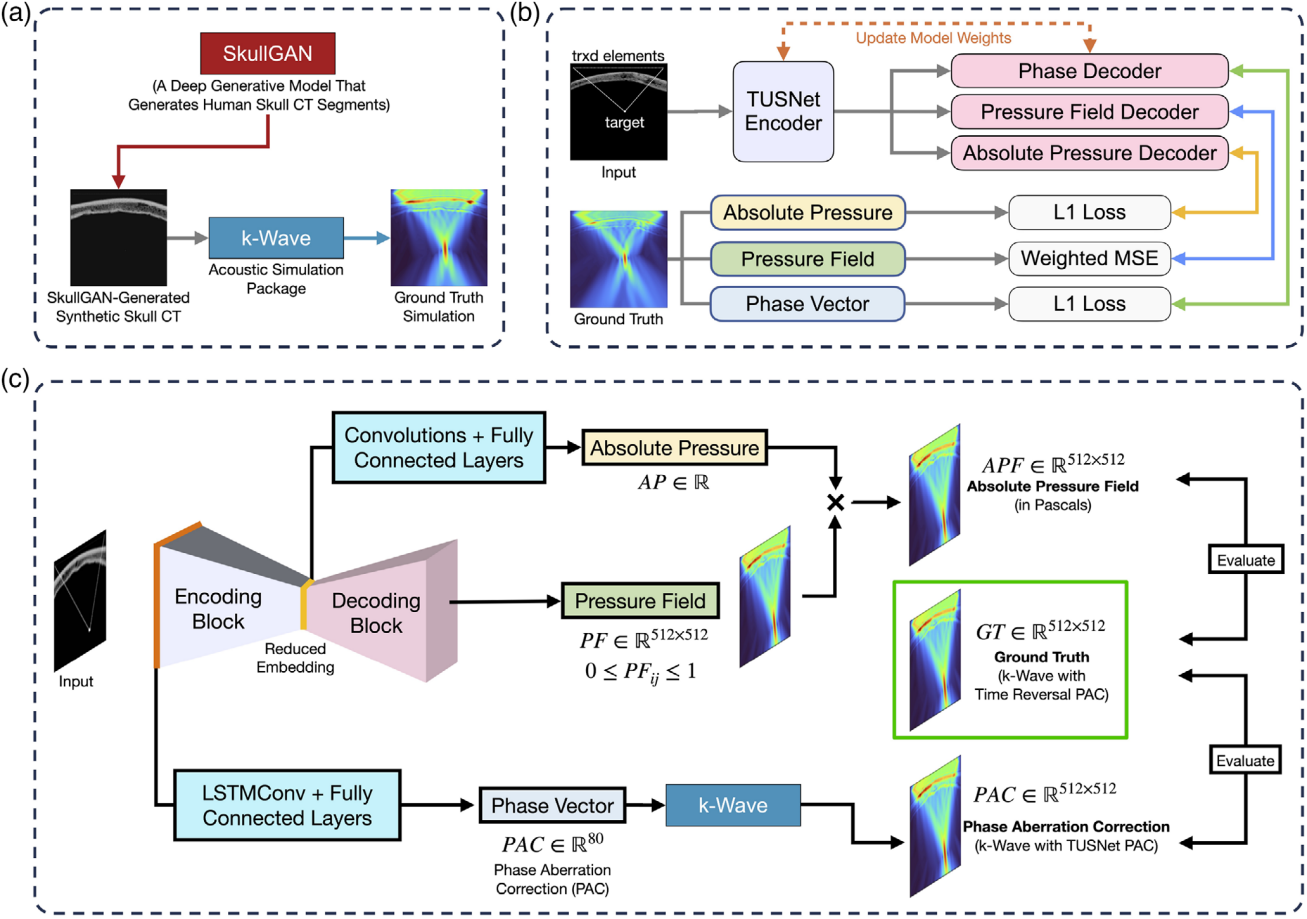
TUSNet training (a‐b) and inference (c) pipelines. (a) TUSNet is trained entirely on synthetic skull CT images generated by SkullGAN, a generative model that, by learning the underlying data generating distribution of real human skull CTs, produces synthetic 2D skull CT segments. Transcranial ultrasound propagation is simulated through the skull using k‐Wave to generate the ground truth training data. (b) TUSNet comprises a multi‐task encoder‐decoder architecture, with three decoders individually predicting the ultrasound *pressure field*, *phase vector* (phase aberration corrections), and *absolute pressure* (peak focal pressure), given an input. The input comprises the skull CT segment, target location, and locations of the transducer elements lying above the skull segment. We first trained the *TUSNet Encoder* and *Pressure Field Decoder* to predict the ultrasound pressure field, after which the *TUSNet Encoder* was frozen and the *Phase Decoder* and *Absolute Pressure Decoder* were trained independently, each using a separate loss. (c) At inference, the *Absolute Pressure* (∈R) was derived from the the *Reduced Embedding*, and the normalized *Pressure Field* from the *Decoding Block*. These were multiplied to produce the *Absolute Pressure Field* in Pascals. The *Phase Vector* was estimated by applying *LSTMConv* and fully connected layers to the *Encoding Block*'s first output, and evaluated with k‐Wave simulations. CT, computed tomography; LSTM, long short‐term memory.

In this paper, we propose TUSNet (Figure 1), a machine learning approach designed to bridge this gap between accuracy and efficiency. Leveraging the power of sequence‐based deep learning architectures, TUSNet is the first end‐to‐end model capable of computing the 2D transcranial ultrasound pressure field and phase aberration corrections for an 80‐element phased array transducer within 0.0207 s (one NVIDIA A4000 GPU [Average of 10 runs, 0.062 s on a MacBook Pro w/ M1 Pro]) while maintaining the desired accuracy for focal positioning and pressure recovery. (This claim is supported by a targeted literature search conducted on Google Scholar in June 2024 using the exact query “deep learning phase aberration correction transcranial focused ultrasound,” which yielded no prior publications reporting an end‐to‐end deep‐learning model for transcranial phase‐aberration correction in focused ultrasound.) The TUSNet phase corrections on average recovered 98.3% of the focal pressure (compared to ground truth data from k‐wave, a widely used acoustic simulation package), while maintaining a mean positioning error of 0.18mm. Although TUSNet is a proof of concept in showing the possibility of performing an end‐to‐end transcranial pressure field simulation and phase aberration correction in 2D, it provides the blueprint for its 3D extension. We believe that the introduction of TUSNet, in conjunction with the other proposed models so far,^32^, ^33^, ^34^ will revolutionize the field of transcranial ultrasound by paving the way for development of clinical models that enable real‐time phase aberration correction and pressure field simulations with high accuracies, significantly streamlining patient treatments.

## 2 METHODS

### 2.1 Model

The training and inference pipelines for TUSNet, as well as its overall architecture, are presented in Figure 1. In the training phase (Figure 1a,b), SkullGAN synthesizes diverse 2D skull computed tomography (CT) slices that approximate real human skull morphologies, while k‐Wave simulations provide the corresponding ground‐truth pressure fields and phase aberration corrections. TUSNet's architecture leverages a multi‐scale encoder–decoder design, incorporating convolutional layers and long short‐term memory (LSTM) modules. Multiple loss functions for absolute pressure, pressure field, and phase vector (Figure 1b) make possible the ultimate reconstruction of the absolute pressure field and phase aberration correction vector (Figure 1c).

**FIGURE 2:**
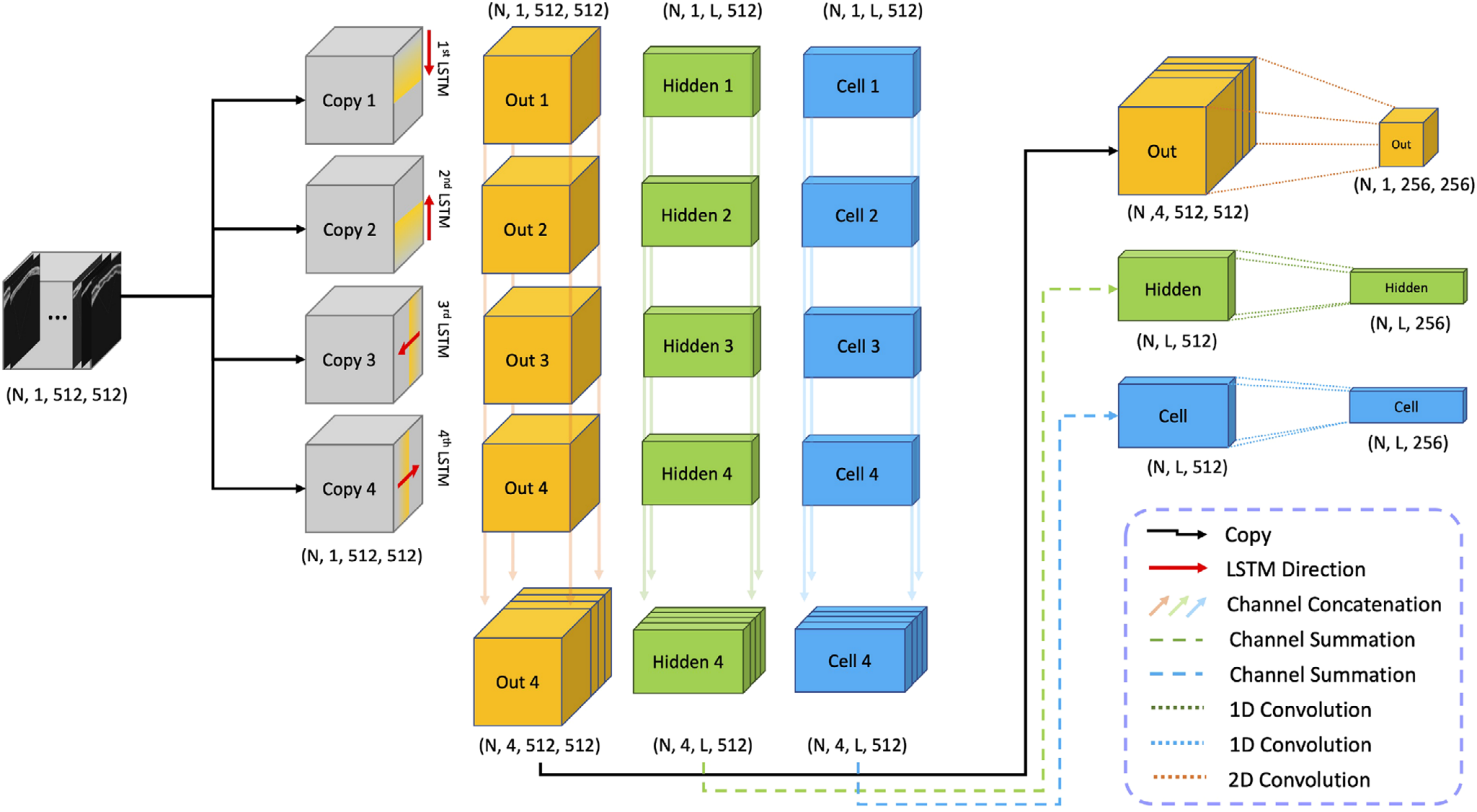
LSTM‐Conv architecture built into the larger TUSNet model. The LSTM‐Conv cell unit consists of four long short‐term memory (LSTM) cells that scan the input images in four directions, as indicated by the red arrows. The outputs of this step are concatenated along their channel dimension and passed through further convolutional layers to reduce the dimensionality of the output. Every LSTM‐Conv cell produces an output, a hidden state, and a cell state, all of which will be used as input to the subsequent LSTM‐Conv cell in a series of five such cells in the encoding block, and five such cells in the decoding block of TUSNet. N refers to the number of samples. LSTM, long short‐term memory.

At the heart of TUSNet lies the LSTM‐Conv cell (detailed in Figure 2), fusing the strengths of LSTM networks with Convolutional Neural Networks (CNN). While LSTMs are generally applied to temporal sequences, prior work has shown that they can also effectively model spatial dependencies in 2D (or higher‐dimensional) data by treating one spatial dimension as a sequential ordering axis. For instance, Liang et al. applied separate LSTM chains along different spatial directions to capture both local and long‐range spatial context across images, achieving state of the art results at the time on semantic object parsing.^35^ Likewise, Hu et al. used a convolutional‐LSTM architecture to jointly model spatial structure and spectral (i.e., band) dependencies in images, preserving spatial organization while exploiting long‐range correlations.^36^ In the medical‐imaging domain, a hybrid MediVision model combined CNNs with LSTM layers to leverage spatial feature extraction followed by sequential context modeling of spatial feature maps, showing improved classification performance over CNN‐only baselines.^37^

Ultrasound pressure fields are a static representation of a temporal process (i.e., the propagation of sounds waves through space)—LSTM layers are therefore suited to capture the spatial, sequential order inherent to ultrasound pressure fields, while the convolutional layers condense this data into compact representations. Each LSTM‐Conv cell initiates with four LSTM units, each scanning the 512×512 input from a different direction (i.e., top–bottom, left–right, and their reverses). These LSTM units have equal hidden sizes that match the input size, and four layers in the final model.

The outputs of these four LSTM units are then concatenated and funneled through two convolutional layers, which alter the dimensionality of the embedding. The first convolutional layer has eight channels and maintains the dimensionality of the input, while the second one expands this to 16 channels while either downsampling or upsampling (corresponding to cells in the encoder and decoder, respectively) the input size by a factor of two.

Finally, a pooling convolutional layer with a 1×1 filter reduces the channel size back to one, ensuring the output matches the required dimensions for the next LSTM‐Conv cell in the model. To enhance the network's stability and learning capability, batch normalization and a Rectified Linear Unit (ReLU) activation function are applied following each convolutional layer, including the final output of the cell. Additionally, a dropout technique with a rate of 0.2 is used to augment the network's robustness and curtail overfitting.^38^

**FIGURE 3:**
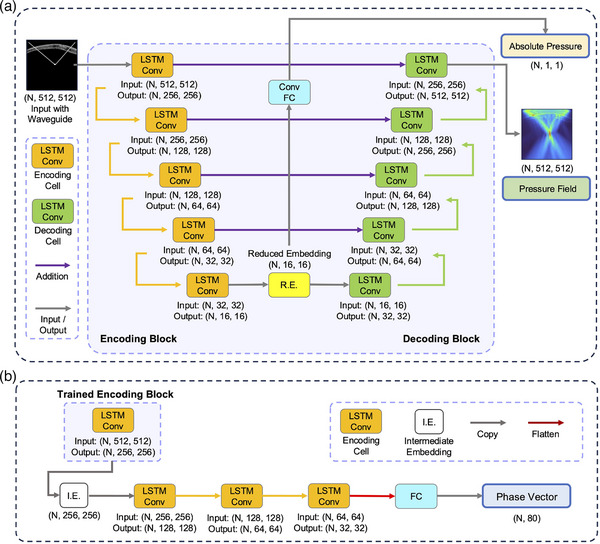
TUSNet architecture. (a) TUSNet consists of a multi‐task encoder–decoder layout, along with skip connections between the two halves of the network. The output of the decoding block is the normalized Pressure Field corresponding to the input skull CT segment and target location. The reduced embedding, R.E., after passing through a series of convolutional and fully connected layers, produces the Absolute Pressure. This scalar value is used to revert back from the normalized Pressure Field to the absolute pressure field in Pascals. (b) The output of the first LSTM‐Conv cell of the trained encoding block is copied to an intermediate embeddings (I.E.). Subsequent series of LSTM‐Conv cells are applied, followed by fully‐connected layers, to yield a vector of length 80, representing the phase corrections for each of the 80 transducer elements. Note that the weights of the LSTM‐Conv cell borrowed from the trained encoding block are frozen and not updated in this process. LSTM, long short‐term memory.

TUSNet is structured as a symmetrical series of LSTM‐Conv cells, as shown in Figure 3a. It is composed of an encoding and a decoding block, each containing five LSTM‐Conv cells, thereby making a total of 10 cells. Starting with the input, the encoding block gradually downsamples the data, transforming and compressing it into a more compact representation. Each LSTM‐Conv cell in this block takes an input size that halves at each successive layer, aligning with the notion of downsampling. Similarly, the decoding block consists of LSTM‐Conv cells, which receive a progressively larger input size, yielding an upsampling process that attempts to rebuild the original data from the condensed representation (i.e., the reduced embedding in Figure 3a).

TUSNet also incorporates skip‐connections,^39^ which link corresponding layers from the encoder and decoder blocks, allowing direct information transfer between these layers. This method is employed to circumvent the issue of information loss due to vanishing gradients during the dense encoding and decoding processes. This approach allows TUSNet to effectively learn compact representations of the skull CT that can be used to efficiently recover the phase aberration corrections required for the transducer, while retaining the level of information required to reconstruct highly detailed and accurate pressure fields.

The phase decoder, described in Figure 3b, uses the trained TUSNet encoding block to reconstruct the optimal phase aberration corrections for a given skull. Initiating this process, the phase decoder passes the output of the first LSTM‐Conv cell of the trained encoding block through three additional LSTM‐Conv layers. This operation downscales the embedding to a lower‐dimensional feature space, while still allowing the model to use more information from the input. After flattening the output of the last LSTM‐Conv cell, a final series of fully‐connected layers are applied to produce the predicted phase delays.

A simpler decoder, using only the reduced embedding produced by the encoder, is used to predict the peak pressure. Given the simplicity of having to estimate only a scalar value (Absolute Pressure in Figure 3a), a series of convolutional and fully‐connected layers applied to the reduced embedding were sufficient in estimating the Absolute Pressure output of TUSNet.

### 2.2 Ground‐truth simulations

All ground‐truth simulations were generated using the MATLAB acoustic simulation package k‐Wave,^40^ a widely used tool in the field. The simulation window was set to 6cm×6cm, or 512×512 pixels, yielding a grid size of 0.117mm. A flat, 80‐element phased‐array transducer with 0.7mm spacing between its elements and total width of 5.5cm was simulated. The transducer was operated at 500 kHz with a pressure of 1 MPa per transducer element, and set to output a 5‐cycle ultrasound pulse over 10μs.

Acoustic properties were determined using the built‐in k‐Wave function hounsfield2density, which applies a linear mapping from Hounsfield Units (HU) to density. The acoustic velocity was calculated using a linear mapping of c=1.33ρ+166.67.^41^ The density was clipped at a lower bound of 997 kg/$m^{3}$ to ensure that areas outside of the skull reflected the density of water. A constant attenuation of 13.3 dB/MHz·cm was applied to the masked skull.^26^, ^42^

Every input to k‐Wave had three components: the 2D skull CT segment, the location of the transducer elements, and the target location. A skull CT slice, represented as a 512×512 array, served as the primary canvas. These skull CT slices were entirely synthetic. In order to train TUSNet, we generated 5000 *synthetic* skull CT slices using SkullGAN, a generative adversarial network that outputs realistic 128×128 synthetic 2D skull CT segments^34^ at 0.625mm in‐plane resolution. Along with allowing the generation of substantial datasets, much larger than those obtainable from real datasets alone, this approach mitigates concerns of privacy as no real patient data was used to train TUSNet. These skull CT segments generated by SkullGAN were highly realistic, as evidenced by the performance of four Stanford staff radiologists who achieved a mean 60% accuracy score in labeling 50 scans (25 of each category) as either real or synthetic. It should be noted that SkullGAN only generated parietal and temporal bones.

After generating the 5000 synthetic slices, to avoid overfitting to a particular skull shape or skewing the training set, the mean squared error (MSE) between each of the possible synthetic pairs was used to filter out similar skull segments and encourage high‐diversity among the slices, which resulted in 3222, 128×128 unique skull segments. After upsampling each synthetic CT slice to 512×512 (using nearest‐neighbor interpolation), we simulated 56 different target locations for each CT slice. This resulted in a total of 3222×56=180432 *synthetic* skull CT simulations to train TUSNet. The training paradigm was supervised, where the input comprised only the skull CT segment, transducer elements, and target location. The corresponding outputs (labels) consisted of normalized phase‐corrected pressure field, absolute pressure scaling factor, and vector of phase corrections.

For testing purposes, similar simulations were run on a dataset of 22 *real* skull CT slices, taken from three completely separate patients, to produce a test set consisting of 22×56=1232 samples (protocol no. IRB 32859). CT slices exhibiting imaging artifacts such as CT streaking artifact, and slices from the frontal and occipital bones that were not represented in the training set, were removed from the test set. As such, roughly 50% of the data was removed when generating the test set and only the temporal and parietal bones were kept. Next, of the remaining skull segments, we randomly sampled 22 of them, which resulted in the final 1232 samples. It should be emphasized that this test set of real skull CTs was never seen either by SkullGAN or by TUSNet.

**FIGURE 4:**
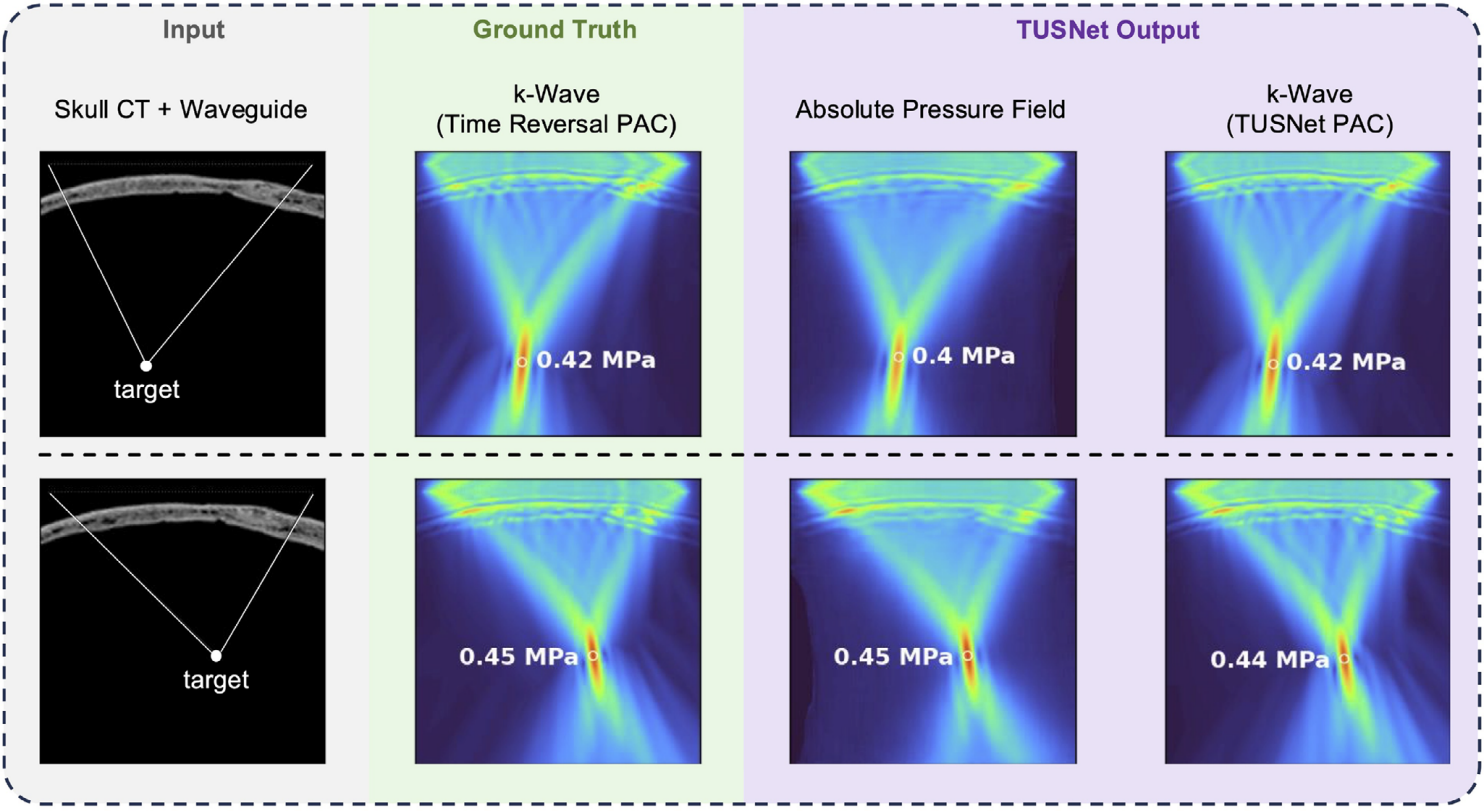
Comparison between ground truth and TUSNet outputs (columns) for two examples (rows). **Input**: consists of the transducer elements above the skull (background removed), a waveguide, and the target. **Ground Truth**: k‐Wave‐simulated ground truth phase aberration‐corrected pressure field using time reversal. **TUSNet Output for Absolute Pressure Field**: in Pascals, rather than normalized values. **TUSNet Output for Phase Vector**: simulated by k‐Wave based on the TUSNet phase vector, rather than time reversal.

The transducer elements were located 3mm from the upper row of this canvas and were denoted by a series of 1's. The location of the target was denoted by a single pixel along with two straight lines connecting the two outermost transducer elements to this single pixel (waveguides, see Figure 4a). These waveguides, computed using the anti‐aliased Bresenham's line algorithm,^43^ depicted the unaberrated propagation paths for the ultrasound waves and helped improve the quality of the simulations. As we examine in the appendix through an ablation study, these waveguides serve to enhance the network's ability and efficiency in identifying the ultrasound propagation path and consequently, the target location.

Next, ground truth phase aberration corrections were computed using time reversal,^44^ wherein an initial simulation was run by sending a test pulse from the intended target to the transducer and recording the receive delay at each element. These phase delays were then applied to the transducer, and the simulation was run forward to produce the steady‐state phase‐corrected pressure field. This sequence had a computation time of about 25.8 seconds per simulation on an NVIDIA A4000 GPU.

These k‐Wave simulations were further processed before being used for training TUSNet: All inputs to TUSNet were normalized to the maximum HU intensity present in the entire dataset of skull CTs. Every output pressure field was normalized to itself, such that they all took values between 0 and 1. These normalized pressure fields, together with their normalization factors (respective peak values), were presented as decoupled targets to TUSNet so that the network could learn to output both the normalized pressure field as well as its corresponding peak value of the pressure. This approach allowed for reconstructing the absolute pressure in Pascals, rather than mere normalized values that bear no physical or physiological importance.

In this numerical study, we have no experimental ground truth for in‐brain pressure fields. Therefore, we adopt the full‐wave time‐reversal solution computed by k‐Wave as the numerical ground truth. This solution represents the highest‐fidelity physical model currently available for transcranial ultrasound propagation and is widely used as a reference in the field. Accordingly, we benchmark TUSNet's performance against this full‐wave time‐reversal solution. For completeness, we also compute geometric ray‐tracing predictions on the same test set, and those results are provided in the Appendix.

Although k‐Wave is widely used for full‐wave acoustic simulation, it remains an approximation of real‐world ultrasonic propagation. The computational domain assumes linear acoustics, isotropic and homogeneous material properties within each voxel, and a simplified, homogeneous power‐law absorption model characterized by a single attenuation coefficient and exponent, which does not capture spatially varying or anisotropic frequency‐dependent absorption observed in real cranial bone.^26^, ^40^, ^45^ Real cranial bone, in contrast, exhibits strongly anisotropic elasticity as well as trabecular and cortical heterogeneity at sub‐voxel scales, producing mode conversion and complex scattering patterns that are not represented in the numerical grid.^46^, ^47^ The governing equations also assume no mode conversion into shear waves in fluid regions and do not fully capture microscopic scattering from fine trabecular structures beyond the grid resolution. While the perfectly matched layer boundary conditions effectively prevent artificial reflections at the outer edges of the computational grid, the simulation domain excludes several real anatomical layers—skin, subcutaneous fat, muscle, meninges, cerebrospinal fluid, and brain tissue with their true acoustic properties—thereby omitting the complex impedance transitions that occur in vivo along the full transcranial path.^48^, ^49^ As a result, the simulated pressure fields represent a high‐fidelity numerical surrogate of propagation, but remain smoother, more linear, and less heterogeneous than true in‐brain fields.^50^ These approximations define the limits of the ground truth used for training and benchmarking TUSNet.

### 2.3 Training

TUSNet's training was structured in a sequential manner, aiming to separately minimize the loss for each task. First, the encoder/decoder architecture was trained to generate normalized ultrasound pressure fields (*Pressure Field* in Figure 3a). Subsequently, as shown in Figure 3a,b, the embeddings produced by the trained encoder were used to train the other arms, to decode the phase corrections (Phase Vector) and simulated peak absolute pressure (*Absolute Pressure*). Because we did not use a physics‐informed loss function, these three tasks were effectively decoupled from one another and the only shared parcel of information among them was contained in the encoding block.

$$\text{Pressure Field (PF)}\in {\left\{0,1\right\}}^{512\times 512},$$

(1) $$\begin{array}{cc} \ell_{\text{PF}} & =\frac{1}{512^{2}}\sum_{i=1}^{512}\sum_{j=1}^{512}W_{ij}{\left({\hat{\text{PF}}}_{ij}-{\text{PF}}_{ij}\right)}^{2}\;w/\;W_{ij}\left(t\right)\\ & =\left\{\begin{array}{cc} \lambda_{\text{skull}}\left(t\right), & i<180,\\ \lambda_{\text{focus}}\left(t\right), & i\geq 180 \end{array}\right. \end{array}$$

$$\lambda_{\text{skull}}\left(t\right)=\left\{\begin{array}{cc} 0.5, & t<5,\\ \frac{0.5}{t-4}, & t\geq 5, \end{array}\right.\;\lambda_{\text{focus}}\left(t\right)=1-\lambda_{\text{skull}}\left(t\right)$$

where $W_{ij}$ is an adaptive weighting factor that emphasizes the focal region over the rest of the field:

Absolute Pressure (AP)∈R,

(2) $$\begin{array}{c} \ell_{\text{AP}}=\left|\hat{\text{AP}}-\text{AP}\right|. \end{array}$$

$$\text{Phase Aberration Correction (PAC)}\in R^{80},$$

(3) $$\begin{array}{c} \ell_{\text{PAC}}=\frac{1}{80}\sum_{k=1}^{80}\left|\hat{{\text{PAC}}_{k}}-{\text{PAC}}_{k}\right|. \end{array}$$

All training was performed on an accelerator‐optimized Google Compute Platform instance with 4 × NVIDIA A100 80GB GPUs. The model was trained using the Adam optimizer with an initial learning rate of 2e‐4. The learning rate was scheduled to decrease by a factor of 0.1 when the validation loss ceased to improve over a patience of 2 epochs, with a threshold of 1e‐3.

Transfer learning and fine‐tuning were also employed to improve the performance of the final model: Initially, TUSNet was trained to convergence with 1 layer in each LSTM. Upon the completion of this preliminary training, the weights from this single‐layered model were transferred to initialize and fine‐tune a more complex 4‐layer LSTM model, which was again trained to convergence. Training was carried out for approximately 50 epochs with a batch size of 256, using a weighted mean squared error (MSE) loss function for the main encoder/decoder, and L1 loss for the phase and absolute pressure decoders. The weighting aimed to enhance the focus's significance, ensuring the model prioritized achieving accurate focus in terms of pressure and location. This approach allowed the model to allocate more resources to on‐target accuracy, even if it meant sacrificing some precision in estimating off‐target pressures, which are less critical due to their negligible energy deposition. Early stopping was also employed as needed to prevent overfitting.

### 2.4 TUSNet output

The *Pressure Field*, 512×512, is the normalized phase corrected field, solely tasked with providing a visual to the target shape, location, and wave distortions. The *Absolute Pressure*, on the other hand, is the scalar multiplier that converts these normalized pressure values to their absolute values, making it possible to retrieve the actual pressure at the target in Pascals. Together, they provide a complete picture: **Absolute Pressure Field** = *Pressure Field* × *Absolute Pressure* (Figure 1c).

The *Phase Vector*, is the list of the 80 time delays that should be applied to each of the 80 transducer elements in order to correct for phase aberrations caused by the skull. To avoid confusion, we refer to this phase vector output as the **Phase Aberration Correction** of TUSNet. In our analysis, we used k‐Wave for transcranial simulations, and compared the pressure field simulated using the *Phase Vector* delays to the ground truth simulated with time reversal^44^ (Figure 1c).

### 2.5 Evaluation metrics

All evaluation metrics were computed as deterministic descriptive measures of performance (means ± standard deviations over the test set). No hypothesis testing was performed, and therefore no corrections for multiple comparisons were required. A variety of metrics were used to assess the performance of TUSNet, calculated for both the TUSNet pressure field and the k‐Wave simulations of the pressure field with TUSNet's phase aberration corrections. The latter served to evaluate the accuracy of phase aberration correction performance of TUSNet, relying on k‐Wave as a proxy for the real transducer (Figure 1c).

The evaluations involving the focal area and focal overlap entailed generating binary masks of ellipses fit to the pressure fields at full width at half maximum (FWHM) of the pressure, using Python's OpenCV package.

Let P be the pressure field and $P_{max}$ be the peak pressure. This ellipse‐shaped binary mask, M, satisfied the following:

$$M\in {\left\{0,1\right\}}^{512\times 512}$$

(4) $$\begin{array}{c} M_{i,j}=\left\{\begin{array}{cc} 1 & \text{if}\;P_{i,j}\geq 0.5\times P_{\max},\\ 0 & \text{otherwise.} \end{array}\right. \end{array}$$

**Focal area error**: To assess the accuracy of the predicted focus area, we computed the absolute difference in the area between the predicted ($M_{\text{pred}}$) and ground truth ($M_{\text{gt}}$) masks, normalized by the ground truth area:

(5) $$\begin{array}{c} \text{Focal Area Error}=\frac{|M_{\text{pred}}-M_{\text{gt}}|}{|M_{\text{gt}}|}\times 100. \end{array}$$

**Focal overlap**: The Intersection over Union (IoU) quantified the overlap between the predicted ($M_{\text{pred}}$) and ground truth ($M_{\text{gt}}$) focal spots. It was given by:

(6) $$\begin{array}{c} \text{IoU}=\frac{M_{\text{pred}}\cap M_{\text{gt}}}{M_{\text{pred}}\cup M_{\text{gt}}}\times 100. \end{array}$$

**Peak pressure error**: The error between the peak pressure predicted by TUSNet ($P_{pred}^{p}$) and the ground truth ($P_{gt}^{p}$) was calculated as:

(7) $$\begin{array}{c} \text{Peak Pressure Error}=\frac{|P_{\text{pred}}^{p}-P_{\text{gt}}^{p}|}{P_{\text{gt}}^{p}}\times 100. \end{array}$$

It must be noted that this peak pressure need not be the pressure at the focus. The peak pressure is taken as the maximum pressure value in the entire simulation window. It may or may not be the same as the pressure at the focus of the transducer (Figure 6).

**FIGURE 5:**
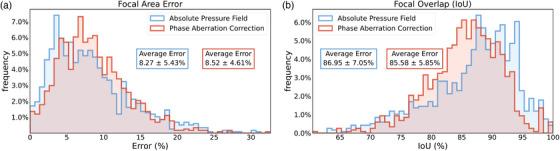
TUSNet performance for the absolute pressure field and phase aberration correction on estimating the focal area for 1232 skull segments. Errors are calculated with respect to time reversal. Focal areas were segmented out as ellipses at the FWHM of the pressure. (a) Frequency distributions (mean and standard deviation values in boxes) of focal area error, evaluated by comparing the areas of the segmented focal spots. (b) Frequency distributions (mean and standard deviation values in boxes) of focal overlap, evaluated by intersection over union for the segmented focal spots.

**FIGURE 6:**
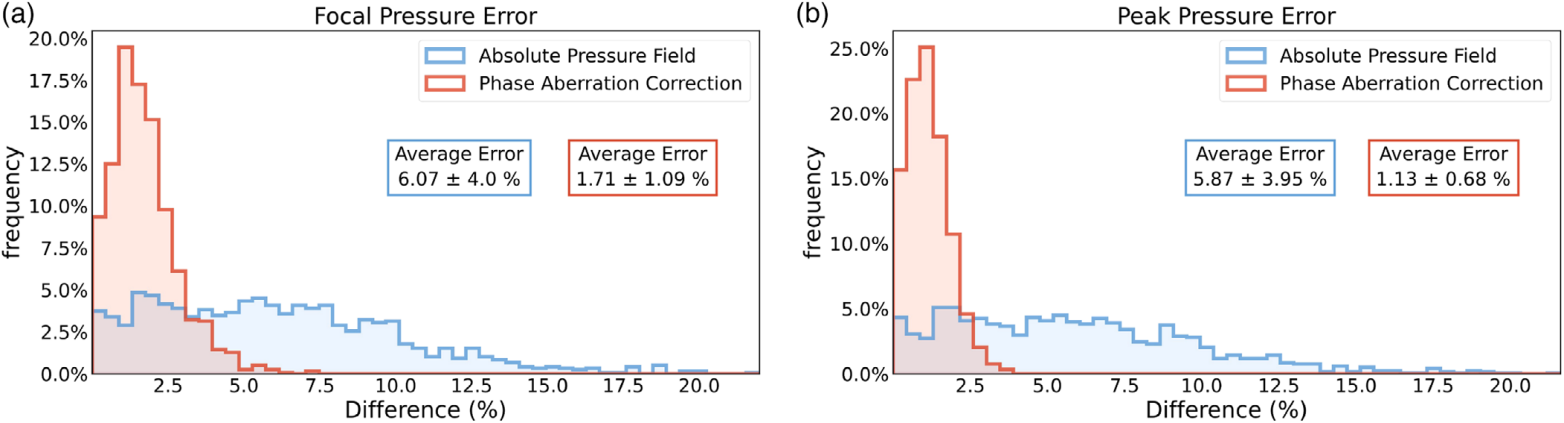
TUSNet performance for the absolute pressure field and phase aberration correction on estimating the focal and peak pressures for 1232 skull segments. Errors are calculated with respect to time reversal. (a) Frequency distributions (mean and standard deviation values in boxes) of the focal pressure error, evaluated by comparing the pressure values at the foci. (b) Frequency distributions of the peak pressure error (mean and standard deviation values in boxes), evaluated by comparing the peak pressures anywhere in the simulation frame.

**Focal pressure error**: The percent error between the focal pressure predicted by TUSNet ($P_{\text{pred}}^{f}$) and the ground truth ($P_{\text{gt}}^{f}$) was calculated using the percent error formula:

(8) $$\begin{array}{c} \text{Focal Pressure Error}=\frac{|P_{\text{pred}}^{f}-P_{\text{gt}}^{f}|}{P_{\text{gt}}^{f}}\times 100. \end{array}$$

Unlike the peak pressure error, the focal pressure error evaluated the difference in pressure at the location of the ground truth pressure field's focus (as determined by the coordinates of the intended point target).

**Focal position error – euclidean distance**: Given the coordinates of the predicted ($f_{\text{pred}}$) and ground truth ($f_{\text{gt}}$) foci, we computed their Euclidean distance in mm as follows:

(9) $$\begin{array}{cc} \text{Euclidean Distance} & =\left[{\left(f{\left(x\right)}_{\text{pred}}-f{\left(x\right)}_{\text{gt}}\right)}^{2}+\right.\\ & {\left.{\left(f{\left(y\right)}_{\text{pred}}-f{\left(y\right)}_{\text{gt}}\right)}^{2}\right]}^{1/2} \end{array}$$

**Focal position error – modified Hausdorff distance**: This metric gauged not only the accuracy in pinpointing the focus, but also the similarity in shape and orientation of the prediction and ground truth full width at half maximum (FWHM) ellipses. The Modified Hausdorff Distance (MHD)^51^ enjoys other advantages such as robustness to noise and applicability to focal spots of different sizes. Let $B_{\text{pred}}$ and $B_{\text{gt}}$ be the boundaries of the largest connected components in the predicted and ground truth pressure fields, respectively. The MHD was then calculated as:

(10) $$\begin{array}{cc} \text{MHD}=\max\{ & \frac{1}{|B_{\text{pred}}|}\sum_{b\in B_{\text{pred}}}\min_{g\in B_{\text{gt}}}d\left(b,g\right),\\ & \frac{1}{|B_{\text{gt}}|}\sum_{g\in B_{\text{gt}}}\min_{b\in B_{\text{pred}}}d\left(g,b\right)\}. \end{array}$$

where d(·,·) denoted the Euclidean distance. $b\in B_{\text{pred}}$ and $g\in B_{\text{gt}}$ represent the points on the boundaries of predicted and ground truth pressure fields, respectively.

**Axial focal position error**: Using the major and minor axes of the FWHM ellipses, we were able to calculate the tilt angle of the focal spots (θ). θ to the right of the normal was considered negative, and θ to the left of the normal was considered positive. This allowed us to project the Euclidean distance in the original Cartesian coordinate system ($d_{x}$ and $d_{y}$) to the major and minor axes of the FWHM ellipse of the predicted field. Specifically, projecting onto the major axis of the predicted FWHM ellipse resulted in the axial focal position error:

(11) $$\begin{array}{c} \text{Axial Error}=d_{y}\cos\left(\theta \right)-d_{x}\sin\left(\theta \right). \end{array}$$

**Lateral focal position error**: Similarly, the lateral component of the focal position error, as projected onto the minor axis of the FWHM ellipse of the predicted field, was obtained using:

(12) $$\begin{array}{c} \text{Lateral Error}=d_{y}\sin\left(\theta \right)+d_{x}\cos\left(\theta \right). \end{array}$$

## 3 RESULTS

The TUSNet outputs, consisting of the normalized pressure field, peak absolute pressure, and phase aberration corrections, are mostly decoupled with varying degrees of parameter sharing between them. As described in Figure 1c, the normalized pressure field utilizes the entire encoding and decoding blocks; the absolute pressure re‐scaling factor shares only the encoding and reduced embedding parameters with the normalized pressure field; and the phase vector arm of TUSNet shares only the first LSTM‐Conv cell of the encoding block. The product of the normalized pressure field and the absolute pressure re‐scaling factor yields the *absolute pressure field* output of TUSNet. The phase vector output of TUSNet is simply an 80‐dimensional vector, corresponding to the corrections applied to each of the 80 transducer elements. In order to be able to evaluate this phase vector with the same criteria and methods used for evaluating the *absolute pressure field*, we implemented transcranial k‐Wave simulations with the TUSNet phase vector, instead of the ground truth method of time reversal, and labeled this output as the *phase aberration correction* output of TUSNet. As such, we present our results separately: for every metric, we evaluated the *absolute pressure field* and *phase aberration correction* of TUSNet independently. End‐to‐end training of the full TUSNet architecture took approximately 8 h on 4× NVIDIA A100 80 GB GPUs, while inference took 21 ms on one NVIDIA A4000 GPU.

### 3.1 Qualitative accuracy

Capturing the shape and location of the focal spot was not the sole objective in training TUSNet. The entirety of the wave pattern, including the side lobes as well as the hot spots inside the skull, was also replicated. Figure 4 showcases the quality of TUSNet's output by comparing them to the ground truth from the k‐Wave simulation. Both the absolute pressure field and the phase aberration corrections estimated by TUSNet successfully replicated the shape and amplitude of the pressure field near the focus while also capturing finer reflections in the skull and outside of the focal spot.

### 3.2 Quantitative accuracy

#### 3.2.1 Focal area

##### 3.2.1.1 Percent error:

The full width at half maximum (FWHM) of the absolute pressure field estimated by TUSNet deviated from that of the ground truth by 8.27%. TUSNet's phase aberration correction had a similar performance at 8.52%. These results are summarized in Figure 5a.

##### 3.2.1.2 Intersection over union (IoU):

On average, the TUSNet absolute pressure field had an 86.95% IoU. The phase aberration correction component of TUSNet did not perform too differently, with a mean IoU of 85.58% (Figure 5b). Previous work on using deep learning to estimate transducer location and orientation for a given, binarized, target ellipsoid, reported 74.49% in IoU.^33^ Another earlier work employing a super‐resolution neural network to convert low resolution (1mm) transcranial ultrasound pressure fields to high resolution (0.5mm), reported an 80.87% IoU.^32^ These studies were in 3D, but neither of them corrected for phase aberrations and used only single‐element transducers.

#### 3.2.2 Pressure

##### 3.2.2.1 Focal pressure error:

The absolute pressure field estimated by TUSNet had a mean error of 6.07% at the focus, compared to the numerical value of the ground truth pressure simulated by k‐Wave. The phase aberration correction of TUSNet incurred only a 1.71% average error (Figure 6a). To our knowledge, this remarkable 98.3% accuracy in recovering the pressure at the focus has no parallel in our field, as other deep learning attempts have only trained on normalized pressure values. HAS recovered 86% of the pressure at the target,^29^ but it was a numerical simulation algorithm, not an end‐to‐end deep learning model. As such, it would still grapple with the accuracy‐efficiency tradeoff mentioned earlier if executed at the 0.117mm grid resolution of TUSNet.

##### 3.2.2.2 Peak pressure error:

Similar to the focal pressure error, the phase aberration correction accuracy of TUSNet outperformed its absolute pressure field in terms of peak pressure error, with a mean error of 1.13% compared to 5.87% (Figure 6b).

#### 3.2.3 Focal position

##### 3.2.3.1 Euclidean distance:

**FIGURE 7:**
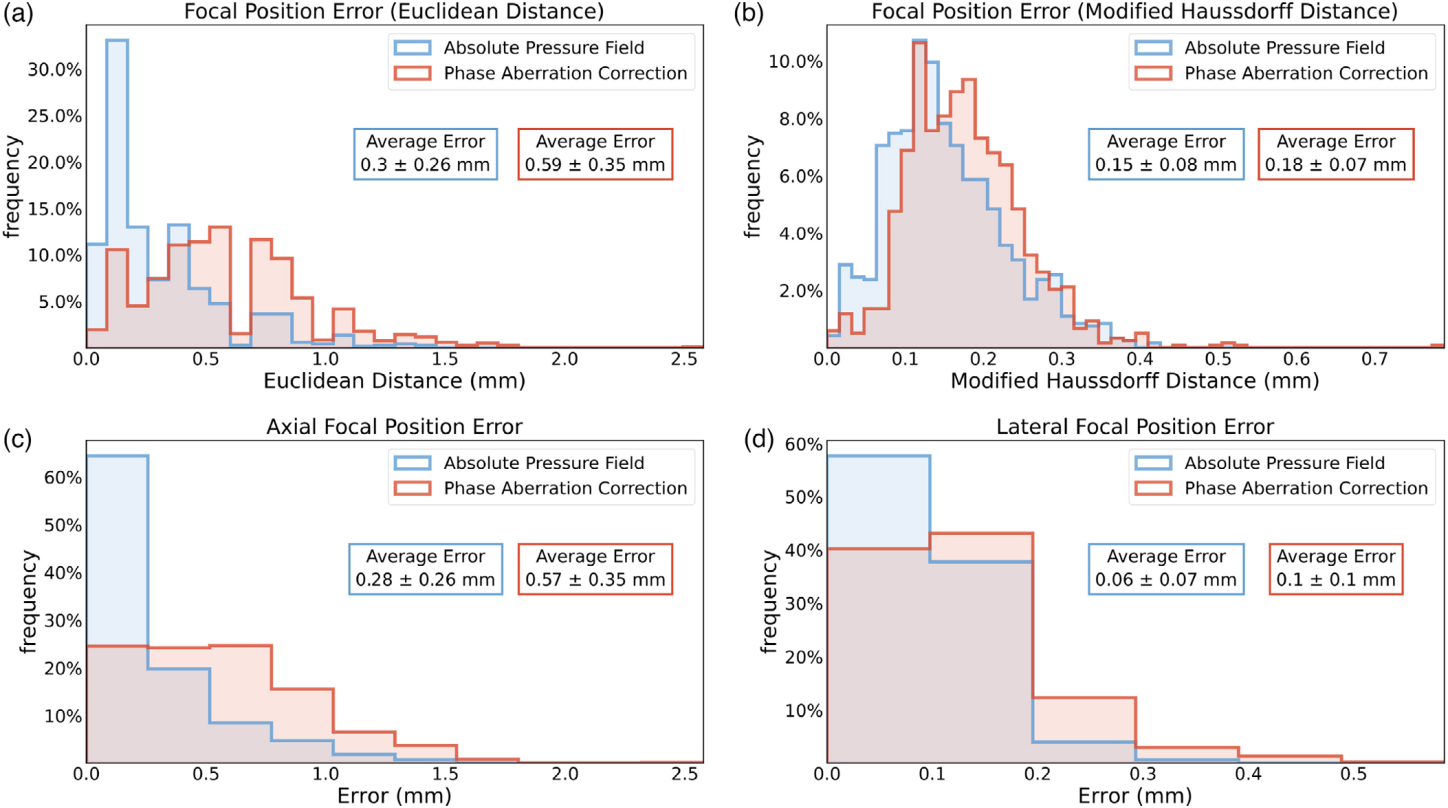
TUSNet performance for the absolute pressure field and phase aberration correction on estimating the focal position for 1232 skull segments. Frequency distributions (mean and standard deviation) values in boxes. Errors are calculated with respect to time reversal. (a) Focal position error, evaluated by comparing the Euclidean distance between the foci. (b) Focal position error, evaluated by the modified Hausdorff distance between the segmented ellipses at FWHM of the foci. (c) Axial focal position error, determined by measuring the distance along the major axis of the FWHM ellipsoid. (d) Lateral focal position, determined by measuring the error along the minor axis of the FWHM ellipsoid. FWHM, full width at half maximum.

Computing the Euclidean distance between the single point with the peak pressure in TUSNet's outputs and k‐Wave ground truth simulations resulted in a mean distance of 0.3mm for the absolute pressure field, and 0.59mm for the phase aberration correction (Figure 7a). Choi et al. reported a 0.96mm transcranial positioning error.^33^ We note that Euclidean distance does not take into account the location and contour error of the entire focal spot, unlike the Modified Hausdorff Distance.

##### 3.2.3.2 Modified Hausdorff distance (MHD):

TUSNet's absolute pressure field was able to achieve a mean of 0.15mm in focal positioning error, as determined with the MHD.^51^ The phase aberration correction had a similar accuracy, scoring an average MHD of 0.18mm. These results are shown in Figure 7b.

##### 3.2.3.3 Axial distance:

As shown in Figure 7c, TUSNet's focal positioning errors were larger in the axial direction compared to the lateral direction, both in the absolute pressure field and phase aberration correction. The former had a mean axial error of 0.28mm, whereas the latter had a mean axial error of 0.57mm.

##### 3.2.3.4 Lateral distance:

Compared to the axial error, the lateral positioning errors of TUSNet were much smaller. The absolute pressure field of TUSNet had a mean lateral error of 0.06mm, while its phase aberration correction had a mean lateral error of 0.1mm (Figure 7d).

This stark difference between the axial and the lateral errors was primarily due to the fact that the axial beam profile is long and, within the tolerance of the model's loss function, several on‐axis points immediately surrounding the focus also qualify as the peak pressure value. Conversely, because the lateral beam profile is much narrower in comparison to the axial beam profile, there is not much room for deviation from the true target location while maintaining a low loss value.

#### 3.2.4 Error analysis of unseen targets

**FIGURE 8:**
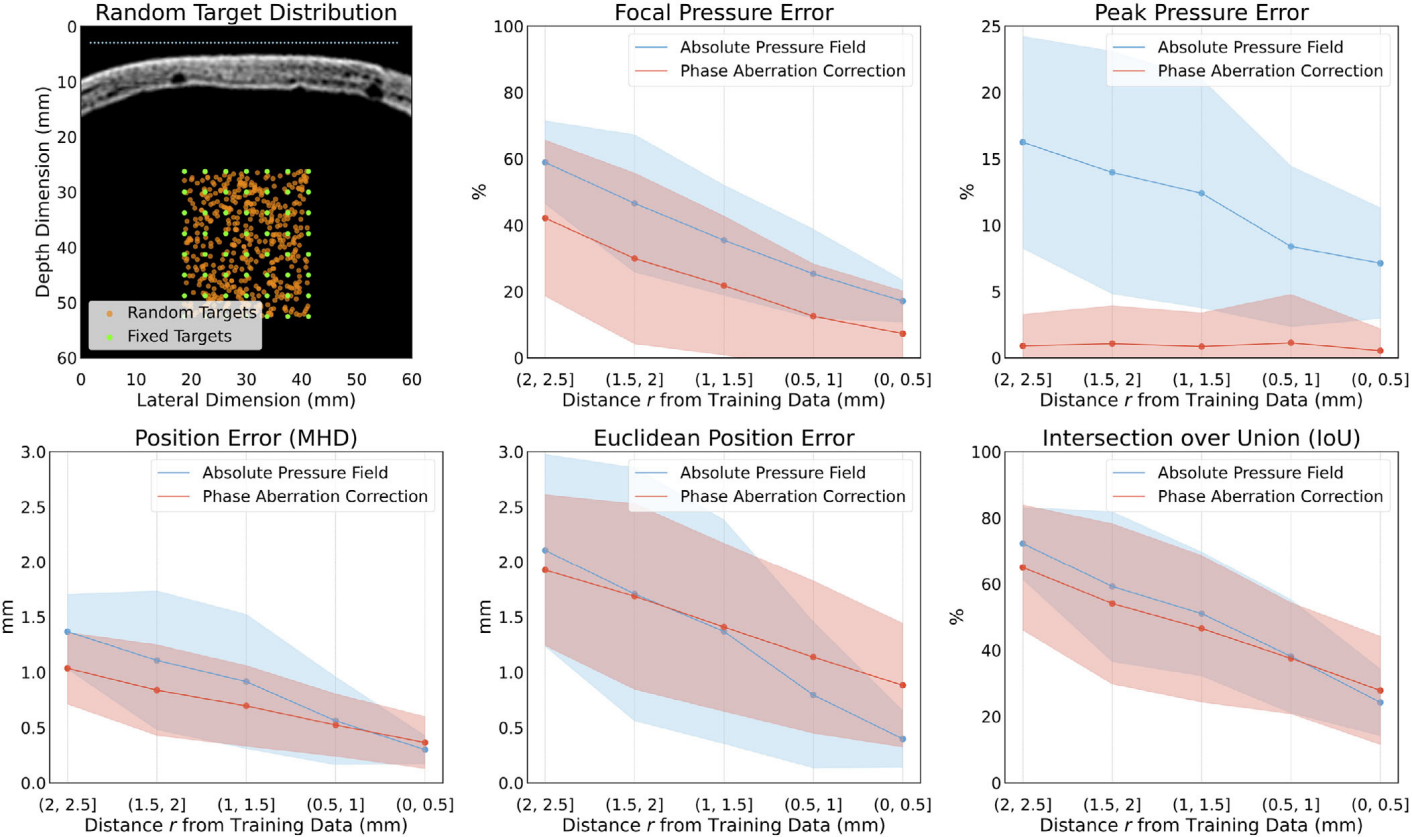
TUSNet performance for the absolute pressure field and phase aberration correction on 500 unseen random test targets. (a) The distribution of fixed training targets (green dots) as well as the distribution of the random test target points (orange dots). The blue dots on top of the real skull CT segment, taken from the test set, are the transducer elements. The 500 random test targets in orange are evaluated across 10 different skull segments, 50 targets per skull. Here, we show only one of those real skull CT segments, but with all of the 500 test targets. (b–f) Error (% or mm) as a function of radial distance of these random targets from the fixed training targets. These distances are in the form of disks, with their inner and out diameters specified along the x‐axis. CT, computed tomography.

In training this version of TUSNet, our emphasis was on generalizing the model to unseen and varied skull CT segments. Therefore, the only variable in this setup was the skull CT—the target locations were held fixed. As shown in Figure 8a, the training fixed targets were placed at a distance of 3.74mm from one another, a spacing that is rather coarse for being able to generalize to unseen random targets in between. However, if we chose to place these targets closer to each other, we would not gain an understanding of the model's beam steering capabilities, as the 56 targets would be highly localized to one area. Conversely, if we wanted to keep the bounding box of the targets the same, and increase the number of target points inside it, we would fall short of the compute requirements for training on so many data points. This tradeoff would be alleviated with access to more computational resources.

To analyze TUSNet's performance over randomly selected targets that did not coincide with the training points, as a function of radial distance away from the training points, we tested 50 randomized targets for each of 10 real skull CT segments. The objective was to see whether there was an inverse correlation between error and distance. In Figure 8b–f, we show that as the distance between the random targets and the fixed training targets reduces, errors also go down. This evidence confirms our assertion that if TUSNet is trained on random targets closely spaced from one another (under 0.5 mm), it will generalize to both unseen targets and unseen skull CT segments.

## 4 DISCUSSION

### 4.1 Principal findings

Transcranial ultrasound has emerged as a promising non‐invasive technique with the potential to revolutionize neurotherapeutic interventions across a spectrum of neurological disorders. However, its clinical translation has been hampered by the difficulty of accurately and efficiently modeling ultrasound propagation through the complex anatomy of the human skull.

In this study, we presented TUSNet, the first end‐to‐end deep‐learning‐based approach to phase aberration correction and pressure field simulation, demonstrating near–real‐time performance (0.0207 s per slice) while maintaining high accuracy, as supported by our quantitative results.

Specifically, TUSNet achieved: Focal area errors of 8.27% (absolute pressure field) and 8.52% (phase correction), with IoUs of 86.95% and 85.58%, respectively (Figure 5, Tables 1 and 2); focal pressure errors of 6.07% (absolute) and 1.71% (phase correction), as shown in Figure 6; sub‐millimeter focal positioning accuracy, with mean Euclidean distances of 0.30 mm (absolute) and 0.59 mm (phase correction), and modified Hausdorff Distances of 0.15 and 0.18 mm, respectively (Figure 7, Tables 1 and 2). These results collectively support the claimed accomplishments of high accuracy, fast inference, and strong agreement with full‐wave ground truth simulations.

**TABLE 1:** TUSNet *absolute pressure field* performance on a test set of real skull CTs.

	Absolute pressure field performance on the test set							
	Focal area error (%)		Pressure error (%)		Focal position error (mm)			
Model trained on	Percent error	IoU (↑)	Focal	Peak	Euclidean	Hausdorff	Axial	Lateral
synthetic CTs	8.27 ± 5.43	86.95 ± 7.05	6.07 ± 4.0	5.87 ± 3.95	0.30 ± 0.26	0.15 ± 0.08	0.28 ± 0.26	0.06 ± 0.07

*Note*: The model was trained on 180 432 SkullGAN‐generated synthetic CTs only. Conversely, the test set included real skull CT segments only that were obtained from three separate patients never seen by either TUSNet or SkullGAN. Errors are calculated with respect to time reversal. ↑ indicates that higher values are better; For all the rest lower values are better.

Abbreviation: CT, computed tomography.

**TABLE 2:** TUSNet *phase aberration correction* performance on a test set of real skull CTs.

	Phase aberration correction performance on the test set							
	Focal area error (%)		Pressure error (%)		Focal position error (mm)			
Model trained on	Percent error	IoU (↑)	Focal	Peak	Euclidean	Hausdorff	Axial	Lateral
synthetic CTs	8.52 ± 4.61	85.58 ± 5.85	1.71 ± 1.09	1.13 ± 0.68	0.59 ± 0.35	0.18 ± 0.07	0.57 ± 0.35	0.10 ± 0.10

*Note*: Phase vectors obtained with TUSNet were input to k‐Wave as the element‐wise phase aberration corrections. The forward simulation results of k‐Wave were used to assess the accuracy of the TUSNet phase vectors against that of time reversal (ground truth). The test set comprised real skull CT segments only, and were obtained from three separate patients never seen by either TUSNet or SkullGAN. Errors are calculated with respect to time reversal. ↑ indicates that higher values are better; For all the rest lower values are better.

Abbreviation: CT, computed tomography.

Prior machine‐learning approaches for transcranial ultrasound simulation have addressed narrower tasks with more constrained assumptions. Shin et al.^32^ used a super‐resolution network to convert low‐resolution finite‐difference time‐domain (FDTD) simulations (1 mm) into higher‐resolution pressure fields (0.5 mm), achieving an IoU of 80.87%, but their model required a physics‐based simulation as input and did not perform phase aberration correction. Choi et al.^33^ predicted optimal location and orientation for a single‐element transducer, but did not reconstruct the pressure field or estimate phase corrections, and their performance was limited to the skull geometries present in their dataset. In contrast, TUSNet performs true end‐to‐end inference from skull CT, target location, and transducer geometry, producing both the pressure field and the 80‐element phase correction vector, with substantially higher IoUs (86%–87%) and sub‐millimeter focal accuracy (Figures 5, 6, 7, Tables 1 and 2). This highlights that TUSNet tackles a more difficult problem while achieving superior accuracy and real‐time inference speed.

One practical barrier the field has long faced is the absence of large, diverse training datasets of human skull CTs. We addressed this challenge using SkullGAN, a generative model capable of producing realistic synthetic 2D skull segments. This allowed us to assemble a dataset of 180 432 transcranial simulations while avoiding privacy concerns associated with real patient data.

**FIGURE A1:**
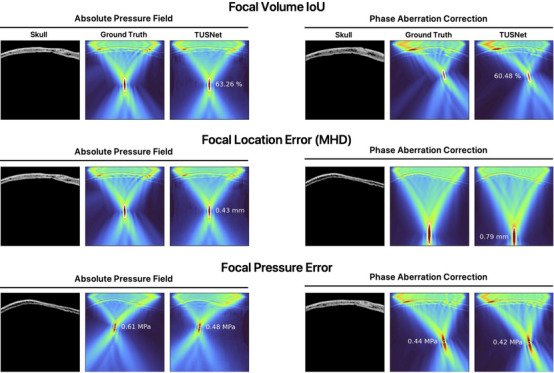
TUSNet worst‐case performance for each of the metrics (focal area, pressure, and positioning error) examined in the paper. The input skull, ground truth (from k‐Wave), and TUSNet output are given for the worst‐performing sample in both the absolute pressure field and phase aberration correction.

TUSNet's strong performance extends beyond the fixed set of 56 training targets. In our randomized target generalization experiment, errors decreased as random targets approached the spatial distribution of training targets (Figure 8), demonstrating that TUSNet can generalize to unseen points in the field of view and motivating future training on more densely sampled target grids. To better understand the model's boundaries, we examined worst‐case performance across metrics (Figure A1) and found that even in the most challenging anatomical cases, positional errors remained under 1 mm, though pressure and IoU metrics degraded in anatomically complex skulls. Furthermore, our skull‐shift analysis showed that small rotations or translations (up to 5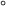 or 4.7 mm) introduce modest performance degradation (Table A5), underscoring the value of incorporating such perturbations during training to improve clinical robustness. Taken together, the results across Figures 5, 6, 7, 8, A1 and Tables 1, 2, A1, A2, A3, A4, A5 support that TUSNet delivers fast, accurate, and generalizable pressure field and phase aberration predictions, establishing a strong foundation for real‐time, patient‐specific transcranial ultrasound simulation.

### 4.2 Limitations

While TUSNet demonstrates strong performance in this 2D numerical setting, several limitations must be acknowledged.

First, the present model is entirely 2D, whereas transcranial ultrasound propagation is fundamentally three‐dimensional. Important physical effects—out‐of‐plane diffraction, 3D trabecular scattering, mode conversion, and complex curvature—are not captured here, and performance in full 3D clinical geometries may differ. Extending TUSNet to 3D will require whole‐skull training datasets, high‐resolution 3D k‐Wave simulations, and substantially larger models.

Second, TUSNet is trained strictly on full‐wave k‐Wave time‐reversal simulations, which, while widely used and considered the closest available numerical proxy to experimental measurements, remain an approximation of true in‐brain acoustics. The model therefore inherits the assumptions and limitations of k‐Wave, including discretization choices, linear acoustic modeling, and idealized boundary conditions. These deviations do not affect the internal consistency of our learning framework, for TUSNet is designed to learn the mapping consistent with the physical model it is trained on, but robust clinical application would require experimental validation to quantify how these numerical–physical discrepancies translate to real‐world TUS performance. Accordingly, deviations from real‐world ultrasound fields arise both from using simulated (k‐Wave) propagation as the supervisory signal and from the approximation inherent in the learned TUSNet mapping itself. Because TUSNet is trained to reproduce the mappings encoded in k‐Wave simulations, it inherits all of the physical approximations of the numerical model. The network learns pressure fields that reflect k‐Wave's assumptions of linear, isotropic acoustics, voxel‐level homogeneity, simplified viscoelastic losses, and limited spatial resolution of fine trabecular microstructures. While this enables excellent internal numerical consistency, it also means that TUSNet cannot recover physical effects that are absent from the training data—such as anisotropic wave speed variations, nonlinear propagation, or shear‐mode interactions in complex bone. Accordingly, the combined numerical–data‐driven system approximates the true ultrasound field only to the extent that these phenomena are negligible. In anatomies or clinical scenarios where such effects dominate, additional training with higher‐fidelity or experimentally measured fields will be required to ensure accurate real‐world performance.

Third, although synthetic training data generated by SkullGAN enabled large‐scale training, the diversity of real skull anatomy exceeds what can be captured by a 2D generative model trained on limited real CTs. As shown in our worst‐case and shift analyses, TUSNet's accuracy decreases for anatomically complex skulls, atypical morphologies, or small misalignments between the skull and transducer. A 3D synthetic generator and broader real CT datasets will be needed to improve robustness.

Fourth, the model was trained on 56 fixed target locations per skull. While the randomized‐target analysis demonstrated promising interpolation behavior, dense training on finer and more uniformly distributed target grids will be required for full, continuous beam‐steering generalization.

Finally, the present framework was trained on one fixed multi‐element transducer geometry—an 80‐element linear array with a fixed placement relative to the skull surface. As such, TUSNet does not yet generalize to alternative array designs, element counts, curvatures, orientations, or placements. Extending the model to multiple array types will require training on expanded datasets that span these geometric variations, and possibly incorporating explicit conditioning on array geometry.

Overall, these limitations show that the present work is a 2D proof of concept, and further development—particularly the transition to 3D, greater anatomical diversity in training data, and improved robustness to shifts and rotations—will be essential for clinical translation.

## 5 CONCLUSION

In this study, we introduced TUSNet, an end‐to‐end deep learning framework for transcranial ultrasound simulation that predicts both the phase‐corrected pressure field and the corresponding 80‐element phase aberration correction vector. Using 180 432 synthetic training samples generated through SkullGAN and full‐wave time‐reversal ground truth, TUSNet achieved high accuracy across all evaluated metrics, including focal area errors of 8.27%–8.52%, focal pressure errors of 6.07% and 1.71%, and sub‐millimeter focal positioning accuracy, as shown in Figures 5, 6, 7 and Tables 1 and 2. The model reproduced key spatial features of the ground‐truth fields, including side lobes and skull‐induced reflections, and its phase corrections yielded accurate full‐wave reconstructions when evaluated with k‐Wave. Importantly, TUSNet performed inference in 0.0207 s, representing more than a 1200× speedup over full‐wave simulation on identical hardware. This result demonstrates that deep neural networks can approximate high‐fidelity, time‐reversal‐based acoustic simulations with strong quantitative agreement while substantially reducing computational cost. Notably, Although these findings indicate strong performance within a controlled numerical environment, the present validation reflects agreement with simulated full‐wave acoustic fields rather than experimental measurements. As such, the reported accuracy should be interpreted within the context of numerical ground truth, with real‐world performance expected to depend on factors such as measurement noise, tissue heterogeneity, and transducer variability. Establishing how well TUSNet generalizes in these experimental settings will be an important direction for future work. Nevertheless, although limited to 2D slices, a single fixed array geometry, and a discrete set of target locations, the present work establishes a viable pathway toward rapid, patient‐specific estimation of skull‐induced phase aberrations and transcranial pressure fields.

Future work will focus on extending TUSNet to three‐dimensional simulations, enhancing its generalization to diverse skull structures and transducer geometries, and integrating it with non‐ionizing imaging modalities such as MRI. These advancements could transform non‐invasive brain therapies, bringing the vision of precision‐focused ultrasound treatments closer to widespread clinical adoption.

## CONFLICT OF INTEREST STATEMENT

The authors declare no conflict of interest.

## Data Availability

TUSNet was written in Python v3.8 using the PyTorch library. All of the source code and training data are available at https://github.com/kbp‐lab/TUSNet.

## A.1 Comparison Against Other Models

In the table below, we summarize the the performance of TUSNet against the other two models referenced in the paper. Direct head to head comparison is difficult, as none of these models tackle the exact same problem under identical conditions. However, a high level comparison of their fixed and variable parameters, outputs, run times, and accuracies gives a sense of the challenges that remain to be addressed.

## A.2 Ablation Study

We made several claims on the superiority of our model architecture to conventional approaches, such as upweighting the loss around the focus compared to other areas of the field of view (weighted MSE), using waveguides rather than point targets to denote the intended target, and the effectiveness of our proposed LSTM‐Conv architecture. In the below table, we compare the performance of TUSNet's absolute pressure field output to its modified variants:

- Mean Square Error (MSE) Loss: under this ablation category, we replaced the weighted MSE with a regular MSE.
- Point Target: under this category, we removed the waveguides from the input and represented the target with only a single point. Waveguides are straight lines connecting the outer extremities of the transducer to the point target.
- Conv‐Only: here, we replaced the LSTM‐Conv cells in the main TUSNet encoder‐decoder with a purely convolutional U‐Net, comprising the same number of parameters as TUSNet (56M).
- Single LSTM: the LSTM‐Conv cells in TUSNet each contain four LSTM layers. For this ablation, we trained a model with only one LSTM layer, thereby giving it a much smaller parameter count of 14M, roughly 1/4 of the full model.
- Uni‐Directional LSTM: each of the four layers in the LSTM‐Conv cells scan the input in a diferent direction (i.e., top–down, down–up, left–right, right–left), after which the output representations are concatenated. Here, while maintaining the same 56M parameter count of the full model, we replaced all of the LSTM with those scanning in only one direction (top–down) to assess the effect of this architectural choice.

The full TUSNet outperformed its modified variants across almost all evaluation metrics. If we were to rank the significance of these different components based on their performance degradation, we would conclude that inclusion of waveguides is the most crucial component, followed by the adoption of a weighted MSE instead of regular MSE loss. Furthermore, the conv‐only architecture struggled significantly, demonstrating that LSTM‐Conv cells provide essential temporal modeling capabilities that enable both superior phase aberration correction and pressure field reconstruction.

Along with ablating components of the main encoder/decoder portion of TUSNet, tasked with predicting the pressure field, we also investigated the impact of using LSTM‐Conv cells in the phase decoder in comparison to simple convolutions. To this end, we saw a significant improvement in TUSNet's phase aberration correction accuracy across all metrics when LSTM‐Conv cells were employed, highlighting the benefits of applying sequence models to this task through our architecture.

## A.3 Comparison Against No Phase Correction and Ray Tracing

In our 2D, 0.5‐MHz numerical benchmark, ray tracing achieved focal accuracy comparable to TUSNet across several metrics. This is expected: in simplified 2D geometries with gentle refraction, relatively uniform bone layers, and limited out‐of‐plane heterogeneity, the assumptions of geometric acoustics hold well. Rays experience minimal multipath behavior, and diffraction effects—poorly handled by geometric methods—remain modest. Under these conditions, both TUSNet and ray tracing approximate the k‐Wave ground truth with low error, but TUSNet does so with substantially lower computational cost. However, these 2D conditions are not representative of realistic scenarios. In 3D skulls, especially at higher frequencies or during off‐axis steering, wave behavior becomes far more complex. Full‐wave studies have repeatedly shown that geometric methods break down in 3D due to (i) strong diffraction, (ii) mode conversion, (iii) multi‐path interference from trabecular structures, and (iv) rapidly varying thickness and curvature of the bone. Prior work demonstrates that ray‐based phase correction accumulates substantial error under these conditions, leading to focal shifts, loss of intensity, and inaccurate phase maps.^29^ TUSNet does not rely on geometric approximations. Because it is trained directly on full‐wave, time‐reversal ground truth, it learns to approximate the underlying wave physics—including diffraction and scattering—that rays cannot capture. As a result, TUSNet retains the accuracy of full‐wave modeling while operating at ray‐like or faster runtimes. Importantly, its performance improves with richer and more diverse training data, allowing it to scale naturally from 2D slices to full 3D skull geometries. Thus, while ray tracing performs surprisingly well in this simplified 2D benchmark, the advantage of data‐driven full‐wave surrogates such as TUSNet becomes decisive as soon as the problem extends to realistic 3D clinical scenarios.

For these reasons, comparisons against ray tracing should be interpreted with caution. Ray tracing is not a physics‐complete model of transcranial ultrasound propagation, and its accuracy breaks down whenever diffraction, scattering, or complex 3D bone structures are involved. As such, agreement with ray tracing does not provide a meaningful assessment of physical accuracy. In purely numerical studies where invasive hydrophone measurements are not available, the only reliable benchmark is a full‐wave solution such as k‐Wave time reversal, which is widely regarded as the closest numerical proxy to experimental ground truth. Accordingly, we use the full‐wave time‐reversal field as the definitive reference in all evaluations, and include ray‐based results only for context in this simplified 2D setting.

## Worst Cases

While TUSNet demonstrated remarkable performance across multiple metrics, there were instances where it underperformed, particularly in cases with high anatomical complexity or poor image quality. These worst cases, while rare, provide crucial insights into the limitations and potential areas for improvement of the model. Here, we examine the worst‐performing samples for each metric evaluated—focal area, pressure, and positioning errors.

In the most challenging cases, TUSNet struggled to replicate the focal area as accurately due to irregular skull geometries that were not well‐represented in the training dataset, achieving an IoU around 60% for both the absolute pressure field and phase aberration correction. A larger number of real skull CTs for training SkullGAN, thereby increasing the diversity of our synthetic skull‐based training set, would help mitigate this problem by better representing diverse skull geometries.

As for positional accuracy, even the worst cases exhibited an error under 1mm. Particularly, for the phase aberration correction worst case, TUSNet's focal spot was inscribed within the ground truth focal spot. However, the Modified Hausdorff Distance reported a 0.79mm error due to the mismatch between the ellipses' shapes.

Pressure estimation presented challenges in cases with pronounced energy loss at the skull, resulting in an unexpected pressure within the brain. The worst case for the absolute pressure field had a focal pressure of 22%, with 7.4% for the phase aberration correction. Additional training on a more diverse population of skull CTs would likely improve these worst‐cases.

## A.4 Shift Analysis

Real‐world clinical applications of transcranial ultrasound require robust modeling that can tolerate slight deviations in skull positioning. Transducer placement, patient movement, and setup variability introduce minor shifts and rotations in skull orientation that can impact ultrasound focusing accuracy. To assess TUSNet's robustness to these shifts, we conducted a shift analysis by applying small spatial transformations—translations and rotations—to a subset of the test skulls and measuring TUSNet's performance under these perturbed conditions.

TUSNet was not trained on any of these transformations or variations, so its performance under these conditions serves as a baseline, with the expectation that accuracy would improve significantly if such shifts were incorporated into the training set. Similar to the randomized targets analysis, this experiment involved selecting five skulls and applying ten random transformations—five shifts and five rotations—across 20 random targets drawn from the training set of 56. The translations consisted of vertical shifts within a range of ±4.7 mm, while the rotations were randomly selected within $\pm 5^{\circ }$ to reflect slight variations in head orientation.

Performance evaluations showed a decrease in focal area accuracy, with the mean intersection over union (IoU) dropping from 86.95% in the standard test set to 79.20% for translations and 79.22% for rotations. This degradation suggests that while TUSNet maintains reasonable focal area predictions under small perturbations, its accuracy does diminish slightly when skull misalignment exceeds a few millimeters or degrees. Absolute pressure predictions showed reasonably low deviation, with focal pressure errors rising from 6.07% to 11.29% at most, with a similar trend for the peak pressure error. Focal positioning accuracy was less impacted, with Euclidean distance errors increasing slightly from 0.30 to 0.41 mm (translation) and 0.42 mm (rotation), and the Hausdorff error from 0.15 to 0.3 mm. Notably, axial positioning errors increased more than lateral errors, reflecting greater sensitivity to skull misalignment along the beam axis.

These results confirm that TUSNet is reasonably robust to small skull shifts but would benefit from explicit augmentation strategies during training to account for real‐world variability. Future improvements could involve incorporating random translations and rotations into the training set, similar to our randomized target tests, to enhance generalization. Additional refinements could include a post‐processing network for dynamic phase aberration correction based on transducer feedback or leveraging multi‐view skull inputs to improve stability in pressure field predictions. While TUSNet already provides a strong foundation for rapid and accurate transcranial ultrasound simulation, these findings highlight areas for further optimization to enhance clinical applicability.

**TABLE A1:** TUSNet performance comparison against SE‐SRResNet, CNN+AC, and k‐Wave.

Model	Input	Output	Dim.	Pressure field	IoU	Positioning error	Focal Pressure Error	Phase Aberration Correction	Generalizes to Various Skulls	Generalizes to Various Target	Inference Run Time	Machine Specifications
SE‐SRResNet ^32^	1.0 mm transcranial simulation (using FDTD algorithm)	0.5mm transcranial pressure field	3D	Normalized	81%	NA	NA	NA	Yes	Yes	3840 ms	one NVIDIA A100 40 GB Tensor Core GPU
CNN+AC^33^	Binarized focal ellipsoid at the desired target	Optimal location for transducer placement	3D	Normalized	74.49%	0.96 mm	NA	NA	No	No	12.25 ms	Intel i9‐7940X‐CPU, 64.0 GB‐RAM, and NVIDIA GeForce 2080Ti‐single GPU
TUSNet	Skull CT segment, target location, and transducer location	Phase corrected transcranial pressure field	2D	Absolute (in Pascals)	85.58%	0.59 mm	1.71%	Yes	Yes	No	20.7 ms	one NVIDIA A4000 16GB GPU
k‐Wave	Skull CT segment, target location, and transducer location	Phase corrected transcranial pressure field	2D	Absolute (in Pascals)	Ground truth	Ground truth	Ground truth	Yes	Yes	Yes	25800 ms	One NVIDIA A4000 16GB GPU

*Note*: The current proof‐of‐concept version of TUSNet is a 2D model, whereas the other two models are in 3D. As such, it is hard to directly compare its superior performance in IoU and positioning error to the other two models. Focal pressure error cannot be compared at all, as the other two models report their final pressure fields in normalized scale. Similarly, the inference run times cannot be compared directly, as the machines on which these models were tested were quite different. However, on a head‐to‐head comparison with k‐Wave, on the same hardware, TUSNet inference time is over 1200 times faster.

**TABLE A2:** TUSNet Absolute Pressure Field performance on the test set under various ablations on the full TUSNet.

	Absolute pressure field performance on the test set							
	Focal area error (%)		Pressure error (%)		Focal position error (mm)			
Ablation:	Percent error	IoU (↑)	Focal	Peak	Euclidean	Hausdorff	Axial	Lateral
MSE loss	10.47 ± 6.22	84.90 ± 7.18	—	—	0.43 ± 0.34	0.19 ± 0.08	0.42 ± 0.35	0.06 ± 0.08
Point target	17.92 ± 16.23	77.04 ± 14.53	—	—	0.35 ± 0.26	0.30 ± 0.20	0.33 ± 0.27	0.06 ± 0.07
Conv‐only	10.80 ± 7.09	79.40 ± 10.37	—	—	0.53 ± 0.5	0.27 ± 0.18	0.51 ± 0.48	0.11 ± 0.16
Single LSTM	7.60 ± 4.83	86.30 ± 6.29	—	—	0.46 ± 0.35	0.17 ± 0.08	0.43 ± 0.35	0.11 ± 0.12
Uni‐Dir LSTM	10.28 ± 8.38	83.90 ± 10.72	—	—	0.37 ± 0.31	0.19 ± 0.13	0.34 ± 0.30	0.09 ± 0.11
Full TUSNet	**8.27 ± 5.43**	**86.95 ± 7.05**	**6.07 ± 4.0**	**5.87 ± 3.95**	**0.30 ± 0.26**	**0.15 ± 0.08**	**0.28 ± 0.26**	**0.06 ± 0.07**

*Note*: *MSE loss* denotes the scenario where we replaced the weighted MSE with a regular MSE. *Point Target* refers to designating the target as a single point without the linear wave guides. *Conv‐Only* is a U‐Net architecture with the same parameter count as the encoder‐decoder component of TUSNet. *Single LSTM* is a version of the model with only one LSTM layer per cell, instead of four, with a total parameter count approximately 1/4 of the full model. *Uni‐Dir LSTM* maintains the same parameter count as the full TUSNet model, but only uses LSTMs that scan in one direction (top‐down) instead of one for each possible direction. *Full TUSNet* denotes the best performing TUSNet model presented in this paper, without any of these ablations. ↑ indicates that higher values are better; For all the rest lower values are better.

Abbreviations: MSE, mean square error; LSTM, long short‐term memory.

**TABLE A3:** TUSNet phase aberration correction performance on the test set under ablation study for the LSTM‐Conv architecture.

	Phase aberration correction performance on the test set							
	Focal area error (%)		Pressure error (%)		Focal position error (mm)			
Ablation:	Percent error	IoU (↑)	Focal	Peak	Euclidean	Hausdorff	Axial	Lateral
Conv‐only	26.14 ± 16.96	60.88 ± 17.75	14.16 ± 14.07	2.98 ± 2.11	1.56 ± 1.10	0.60 ± 0.35	1.39 ± 1.13	0.49 ± 0.43
LSTM‐Conv	**8.52 ± 4.61**	**85.58 ± 5.85**	**1.71 ± 1.09**	**1.13 ± 0.68**	**0.59 ± 0.35**	**0.18 ± 0.07**	**0.57 ± 0.35**	**0.10 ± 0.10**

*Note*:*Conv‐only* denotes the scenario where we used only a network of convolutional and fully‐connected layers to predict the optimal phase aberration correction. *LSTM‐Conv* denotes the TUSNet phase decoder presented in this paper, which employs LSTM‐Conv and fully‐connected layers to estimate the phase aberration correction. ↑ indicates that higher values are better; For all the rest lower values are better.

Abbreviation: LSTM. long short‐term memory.

**TABLE A4:** Comparison of TUSNet and ray tracing to no phase correction.

	Performance on the test set								Runtime (s)	
	Focal area error (%)		Pressure error (%)		Focal position error (mm)				Phase corr.	Full wave
	Percent Error	IoU (↑)	Focal	Peak	Euclidean	Hausdorff	Axial	Lateral		
No phase correction	12.99 ± 6.30	74.80 ± 9.10	4.32 ± 3.05	1.35 ± 1.10	1.04 ± 0.42	0.34 ± 0.13	0.97 ± 0.44	0.28 ± 0.19	NA	14
Ray tracing	4.92 ± 2.79	90.36 ± 4.20	1.95 ± 0.63	1.70 ± 0.57	0.30 ± 0.17	0.11 ± 0.04	0.28 ± 0.18	0.09 ± 0.07	0.04	14
TUSNet	8.52 ± 4.61	85.58 ± 5.85	1.71 ± 1.09	1.13 ± 0.68	0.59 ± 0.35	0.18 ± 0.07	0.57 ± 0.35	0.10 ± 0.10	0.011	0.0207

*Note*: *No Phase Correction* applies the time delays corresponding to free water simulations. *Ray Tracing* draws straight lines from each transducer element to the focus and calculates time of flight based on SOS of each pixel along its path. ↑ indicates that higher values are better; For all the rest lower values are better.

**TABLE A5:** TUSNet pressure field performance with skull transformations.

	Absolute pressure field performance with different skull transformations							
	Focal area error (%)		Pressure error (%)		Focal position error (mm)			
Transformation:	Percent error	IoU (↑)	Focal	Peak	Euclidean	Hausdorff	Axial	Lateral
Translation	7.15 ± 5.62	79.20 ± 9.22	11.29 ± 5.14	10.55 ± 5.18	0.66 ± 0.38	0.30 ± 0.17	0.64 ± 0.38	0.09 ± 0.08
Rotation	7.72 ± 6.07	79.22 ± 9.25	10.16 ± 5.29	9.49 ± 5.29	0.69 ± 0.34	0.30 ± 0.16	0.67 ± 0.34	0.10 ± 0.08
Test set	8.27 ± 5.43	86.95 ± 7.05	6.07 ± 4.0	5.87 ± 3.95	0.30 ± 0.26	0.15 ± 0.08	0.28 ± 0.26	**0.06 ± 0.07**

*Note*: Performance was examined on a set of skulls that were shifted vertically (within 4.7mm) or rotated slightly (within $5^{\circ }$), to reflect real‐world error in transducer placement. Note the test set did not reflect any of these transformations.
